# A Matched Case-Control Study Examining the Association Between Exposure to Depot Medroxyprogesterone Acetate and Cerebral Meningioma Using an Active Comparator

**DOI:** 10.3390/curroncol32070401

**Published:** 2025-07-13

**Authors:** Russell Griffin, Rebecca Arend

**Affiliations:** 1 Department of Epidemiology, School of Public Health, University of Alabama at Birmingham, Birmingham, AL 35294, USA; 2 Department of Obstetrics and Gynecology, Heersink School of Medicine, University of Alabama at Birmingham, Birmingham, AL 35294, USA; rarend@uabmc.edu

**Keywords:** cerebral, meningioma, medroxyprogesterone, levonorgestrel, norethindrone

## Abstract

The recent literature has reported an increased association between the use of depot medroxyprogesterone acetate (dMPA) and cerebral meningioma (CM). Prior studies have been limited in generalizability and did not use an active comparator as a control. The current matched case–control study utilized a bootstrapped sampling design, matching 241 CM cases with controls (i.e., women diagnosed with non-meningioma brain, breast, or skin tumor, one control per type for three total) on age ± 5 years and diagnosis date ± 3 months. Conditional logistic regression was used to estimate odds ratios (ORs) compared with an active (norethindrone or levonorgestrel) and non-active control group. Exposure to dMPA at any time point was not associated with the diagnosis of cerebral meningioma (OR 1.75, 95% CI 0.81–4.95). Exposure to dMPA within a year of diagnosis was associated with the diagnosis of CM compared to both an active control (OR 3.38, 95% CI 1.13–9.70) and a non-active control (OR 6.90, 95% CI 2.31–17.58). This association was also present for those who were exposed within two years prior when compared to a non-active control (OR 3.54, 95% CI 1.50–11.88) but not an active control. Combined with the prior literature, the current results suggest that future research is warranted to understand this association.

## Simple Summary

Research has reported an increased association between injected medroxyprogesterone acetate—a synthetic progesterone used for contraception—and cerebral meningioma, a type of tumor that affects the meninges, which are the covering layers of the brain. To build upon the prior literature, the current study compared medroxyprogesterone acetate exposure to two comparator groups: an active contraceptive comparator (i.e., levonorgestrel and norethindrone) and non-active comparator. Medroxyprogesterone acetate exposure was associated with increased odds of cerebral meningioma for exposure within the prior year compared to both the active and non-active comparator group and for two years prior compared to non-active comparator exposure. The current study supports the prior findings of an increased association between cerebral meningioma and injected medroxyprogesterone acetate exposure; this study adds to the literature that the association persists when compared to an active comparator. Clinicians may want to consider discussing with patients the increased association between injection medroxyprogesterone acetate and cerebral meningioma.

## 1. Introduction

Medroxyprogesterone acetate (MPA) is a synthetic version of progesterone, a sex hormone commonly used as a contraceptive throughout the world. It is used via both an oral route and an injection route, with the former commonly used in combination with an estrogen and the latter route used as the sole ingredient. The injectable route of MPA is known as depot MPA (dMPA) and is used as a contraceptive in the United States by approximately one-quarter of women aged 15–49 [1]. dMPA is administered every three months via either a 150 mg/mL intramuscular injection or a 104 mg/0.65 mL subcutaneous injection. As a contraceptive, dMPA acts on the body by binding to the progesterone receptor of the hypothalamus, which causes the body to prevent ovulation and causes the cervical mucus layer to thicken, making it more difficult for sperm to pass through following intercourse [2].

The recent literature has reported an association between the use of dMPA and the risk of cerebral meningioma [3,4]. Cerebral meningiomas are a common form of intracranial tumors [5], and there is evidence that progesterone plays an active role in the growth of the tumors. These meningiomas have been observed to occur more often among pregnant women [6], and the literature has reported a prevalence as high as 88% of progesterone receptors on meningiomas [7]. In Roland et al. [4], an over five-fold increased risk was reported for cerebral meningioma for those with a dispensation of MPA within the second year prior to the date of meningioma diagnosis. Griffin [3] reported weaker yet still significant 70% increased odds of cerebral meningioma among dMPA users with 3.7-fold increased odds among those who had dispensations of dMPA within the three years prior to the diagnosis date. These studies, however, have been limited by a low prevalence of exposure (i.e., Roland [4]) or the use of a study population that was limited to women who had commercial insurance through their employer (i.e., Griffin [3]); in addition, these studies did not use an active comparator. In light of this, the objective of the current study was to evaluate the association between dMPA and cerebral meningiomas utilizing a control population consisting of women who were not limited to a type of insurance and who were diagnosed with non-meningioma cancer in order to determine if the prior reported association between dMPA and cerebral meningioma persists with the use of a different control group.

## 2. Materials and Methods

### 2.1. Study Design and Population

The study population for the current case–control study consists of women aged 18 or older who were diagnosed with cancer at the University of Alabama at Birmingham, a large academic medical center that serves a population of over one million individuals and that is home to the only National Cancer Institute—designated the comprehensive cancer center in Alabama. This study was reviewed by the institution’s IRB and deemed exempt from IRB review according to the exempt category of “secondary research uses of identifiable private information or identifiable biospecimens”.

For purposes of this study, cases were women who were diagnosed with a cerebral meningioma between 2015 and 2024. Controls consisted of women aged 18 or older who were diagnosed with a malignant breast tumor (e.g., ductal carcinoma), skin tumor (e.g., melanoma), or non-meningioma brain tumor (e.g., glioma). The control groups were chosen to provide a comparison group with a tumor to the same organ as cases (i.e., brain tumor controls), a group with a tumor that is also affected by sex hormones (i.e., breast tumor controls), and a group with a tumor that is not affected by sex hormones (i.e., skin tumor controls). ICD-O-3 topography codes were used to identify meningioma cases (C70), skin tumor controls (C44), non-meningioma brain tumor controls (C71, excluding histology codes beginning with 953), and breast tumor controls (C50). Lists of the first three digits of the histology codes for each group in order of descending frequency are reported in Table 1.

### 2.2. Study Variables

For each study subject, data was collected on age, race, ZIP code, primary insurance type, and ICD diagnosis codes for the three years prior to the diagnosis date up to a month after the diagnosis date. For purposes of analysis, race was recoded as White and Non-White and insurance status as Medicaid or Medicare, commercial insurance, other insurance type (e.g., Champus/TriCare, charity/indigent care), and uninsured. ZIP code was used to identify the subject’s Rural–Urban Commuting Area (RUCA) of residence [8], categorizing the values as metropolitan, micropolitan, and small metro/rural. The ICD diagnosis codes were used to determine Elixhauser comorbidities, from which an unweighted Elixhauser comorbidity score was computed as the count of Elixhauser comorbidities.

Exposure to dMPA, levonorgestrel, or norethindrone was determined through the presence of documented use via medication orders by providers or documentation of use by the subject during medication reconciliation at a medical encounter, whether it be an inpatient or outpatient encounter. Variables were created for any dMPA exposure prior to diagnosis, exposure one year prior to diagnosis, and exposure two years prior to diagnosis. Each matched control was categorized as a non-active comparator or an active comparator; the latter was defined as any documented use of levonorgestrel (in combination with another hormone) or norethindrone (alone or in combination), two commonly used oral contraceptives. Levonorgestrel alone (rather than in combination) is used for emergency contraception rather than continued use and was excluded from exposure consideration due to the short duration of exposure. Non-active comparators were controls who had no documented use of an oral contraceptive.

### 2.3. Statistical Analysis

A conditional logistic regression was used to estimate odds ratios (ORs) and associated 95% confidence intervals (CIs) for the association between dMPA exposure and cerebral meningioma diagnosis. Models were adjusted for age, race, and urban/rural classification of residential ZIP code, insurance type, and unweighted Elixhauser comorbidity score. Separate models were run for each of the dMPA exposure variables (i.e., any exposure, exposure within one year, and exposure within two years) and for each of the three tumor control types. SAS v9.4 was used for all analyses, and *p*-values < 0.05 were considered statistically significant.

### 2.4. Bootstrapping

**Figure 1:**
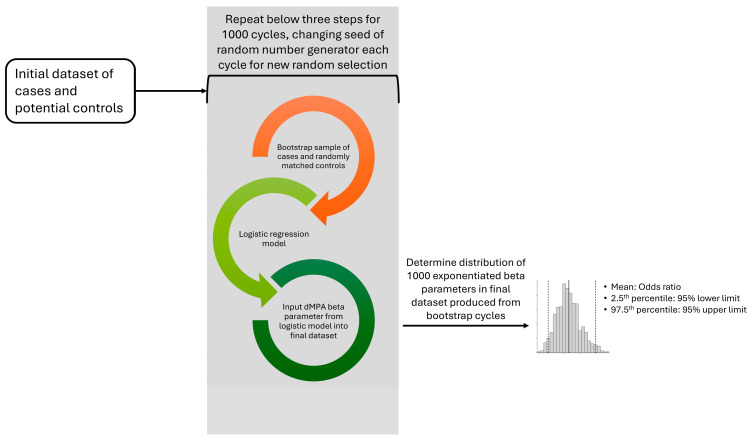
Bootstrap design flowchart. Cases and controls are resampled in 1000 separate cycles with the beta parameter estimate from the logistic regression input into a final dataset. The final dataset of 1000 beta parameters is used to produce the odds ratio (exponentiation of the mean of the beta parameters) and 95% confidence intervals of the odds ratio based on the 2.5th and 97.5th percentiles of the distribution.

The originally intended study design was to perform a 3:1 matching ratio with one control from each group matched (without replacement) to a case based on age ± 5 years and diagnosis date within three months of the case diagnosis date; however, a low prevalence of dMPA exposure among cases resulted in OR estimates that were unstable. In order to produce more stable OR estimations, a bootstrapping method was utilized in which the matching algorithm was performed for 1000 cycles with controls randomly sampled within each cycle (Figure 1). A control could be selected in multiple cycles; however, the control could only be selected once for each cycle.

For bivariate comparisons, among the controls, the frequency of race, RUCA, insurance type, and Elixhauser comorbidity category, and the mean of age and unweighted Elixhauser comorbidity score were calculated for each bootstrap cycle. The pooled estimate was calculated as the mean of the values across the cycles, and 95% CIs of each value were calculated as the 2.5th and 97.5th percentiles of the values. Cases were considered statistically significantly different from controls if the case value fell outside of the pooled control 95% CIs. Similarly, to produce the pooled OR and 95% CIs for an association between dMPA exposure and meningioma, the conditional logistic regression models were run for each of the 1000 matched datasets. The mean of the ORs was calculated as the pooled point estimate, and the 2.5th and 97.5th percentiles were calculated as the lower and upper 95% CI, respectively. In a sensitivity analysis to examine the effects of the control group on OR estimates, models were run, leaving each of the three groups out (e.g., excluding brain tumor controls and keeping melanoma/carcinoma and breast tumor controls in the model).

## 3. Results

A total of 241 cerebral meningioma cases were identified. Most (97.0%) of the matched skin tumor controls were diagnosed with melanoma with a majority having a diagnosis of low cumulative sun damage melanoma (histology code 8743/3, 33.7%), malignant melanoma not otherwise specified (8720/3, 32.7%), or melanoma in situ (8720/2, 21.0%) (Table 1). A majority (89.3%) of non-meningioma brain tumor controls were diagnosed with gliomas; specifically, most were diagnosed with glioblastoma not otherwise specified (histology code 9440/3, 41.1%), astrocytoma (9400/3 and 9401/3, 23.5%), or malignant glioma (9380/3, 6.0%). Lastly, a majority (93.8%) of breast tumor controls were diagnosed with ductal or lobular neoplasms, specifically infiltrating duct carcinoma (histology code 8500/3, 61.2%), non-infiltrating intraductal carcinoma (8500/2, 9.9%), lobular carcinoma (8520/3, 6.6%), or infiltrating duct and lobular carcinoma (8522/3, 5.9%).

The mean age of the cases was 59.6 years; further, cases were slightly over half (58.6%) of white race, mostly (73.6%) lived in a metropolitan area, had commercial insurance (71.4%), and on average had two comorbidities (Elixhauser comorbidity mean score 2.3) (Table 2). Compared to brain tumor controls, cases were older, more likely to be of non-white race, and have Medicare or Medicaid insurance coverage, but cases had a lower Elixhauser comorbidity score (Table 2). When compared to breast tumor controls, cases were more likely to live in a micropolitan area, more likely to be uninsured, and had a higher Elixhauser comorbidity score (mean 2.3 vs. 1.4). Finally, compared to skin tumor controls, cases were far less likely to be white (58.6% vs. 95.3%, 95% CI 93.5–97.0%), less likely to have commercial insurance, and had a higher Elixhauser comorbidity score on average (2.3 vs. 1.2).

Across the bootstrap cycles, 1.1% (95% CI 0.6–1.7%) of controls on average were exposed to dMPA at any point prior to their tumor diagnosis, and 5.4% (95% CI (4.2–6.8%)) were exposed to levonorgestrel combinations or norethindrone. In crude models, any exposure to dMPA was associated with 2.75-fold increased odds of being diagnosed with cerebral meningioma relative to the non-active comparator group (OR 2.75, 95% CI 1.69–5.56) and a two-fold increase when compared to an active comparator (OR 2.08, 95% CI 1.17–4.26). These associations, however, became non-significant in fully adjusted models (active comparator: OR 1.16, 95% CI 0.54–2.95; non-active comparator: OR 1.91, 95% CI 0.99–4.50) (Table 3). Exposure to dMPA within one year of the case date was associated with, in fully adjusted models, an over three-fold increased association being a case (OR 3.27, 95% CI 1.19–9.89) compared to an active comparator and 6.7-fold increased odds of being a case compared to a non-active comparator (OR 6.71, 95% CI 2.69–18.09). For exposure within two years, only the association for dMPA exposure compared to a non-active comparator was significantly increased (OR 3.73, 95% CI 1.75–9.82).

**Figure 2:**
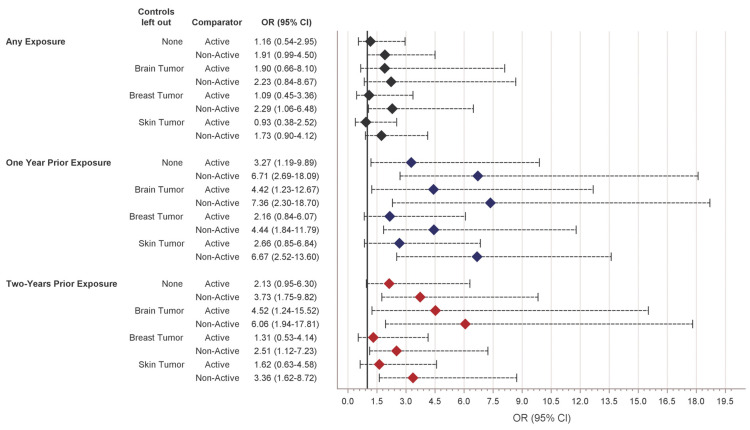
Sensitivity analysis involving a leave-one-out analysis of the association between dMPA and cerebral meningioma based on the exclusion of control groups.

In the leave-one-out sensitivity analysis, associations for the active and non-active comparators were similar across the controls left out (Figure 2). For exposure within one year, associations were stronger for both comparator groups when excluding brain tumor controls. A similar pattern was observed for exposure within two years. For all comparisons, associations within the same exposure period and comparator group were similar across the leave-one-out models.

## 4. Discussion

### 4.1. Principal Findings

In this single-center study investigating the association between dMPA exposure and cerebral meningioma diagnosis, no significant association was observed for any prior dMPA exposure. However, there was a significant association for dMPA exposure compared to a non-active comparator within two years prior to tumor diagnosis. Additionally, exposure within one year was significant when compared to an active and non-active comparator of levonorgestrel or norethindrone. Across tumor control types, the associations were the strongest when brain tumor controls were excluded, and associations were similar in comparisons of cases with the exclusion of either breast tumor or skin tumor controls.

### 4.2. Results in the Context of What Is Known

Prior studies have reported an increased association between dMPA exposure and cerebral meningioma. Roland et al. [4] reported 5.6-fold increased odds of cerebral meningioma among French women who used MPA for the two years prior to their index date. There are some possible reasons why our analysis reports a weaker association. First, dMPA exposure prevalence in the French study was much less than in the current study, with 0.05% of cases and 0.01% of controls with documented exposure. Further, the control group consisted of women who did not have a surgery for cerebral meningioma, and the cases consisted of women who had surgery to treat cerebral meningioma. The current study included any woman with a cerebral meningioma diagnosis as a case, regardless of treatment type, and controls were limited to women with either a non-meningioma brain tumor, breast tumor, or a melanoma/carcinoma.

In a more recent study utilizing a United States population, any exposure to dMPA was associated with a near-70% increased odds of having a cerebral meningioma [3]. Unlike the prior study, associations were strongest in our study among those with dMPA exposure within the year prior to tumor diagnosis. This is likely due to the use of tumor controls in this study, although it is also possible that the difference could be due to the prior study being limited to those who had commercial insurance through their employer.

It is not unexpected that the associations were stronger when excluding brain tumor controls, given that both cerebral meningioma and brain tumors involve the same organ. Thus, it is possible that the meningioma cases and brain tumor controls in our study share similar unmeasured risk or protective factors such as family history [9]; physical activity level [10]; prevalence of allergy [11,12,13], prior hospitalization for epilepsy, diabetes, or stroke [14]. That said, the associations with dMPA exposure in one year remained significant in the current study when brain tumor controls were included in the control groups. Moreover, the associations for each of the leave-one-out models were within the confidence limits of the other two models.

### 4.3. Implications

It has been hypothesized that sex hormones, particularly progesterone, play a role in the oncogenesis of cerebral meningiomas. This hypothesis is further supported by the fact that there has been a reported increase in the size of cerebral meningiomas during pregnancy [6]. Further, the prevalence of progesterone receptors on meningiomas is as low as 38% and as high as 88% [7,15]; this prevalence is greater among meningiomas found as the skull base [16] and—important for the population using progestins—among females [17,18,19], and younger individuals [20]. A recent study reported that medroxyprogesterone acetate had the fourth highest progesterone receptor affinity among 11 tested progestins, being lower than only levonorgestrel, desogestrel, and nomegestrol acetate [21]. One possible biological mechanism of the currently observed association of dMPA exposure with cerebral meningioma could be related to an increased meningioma growth from the presence of medroxyprogesterone acetate and its interaction with the progesterone receptors on the meningioma. Receptor affinity may also explain the lower associations when dMPA was compared to an active control, since levonorgestrel potentially has a higher progesterone receptor affinity. Research has also suggested that progestin-related meningiomas have higher rates of mutations in the PIK3CA gene [22]. These mutations have been associated with decreased apoptosis [23] and have been shown to activate the PI3K/AKT/mTOR pathway, which is potentially involved with tumor progression [24] and has been associated with tumorigenesis of other cancer types [25].

Despite the aforementioned potential mechanism, research is still needed to examine the physiological link to dMPA exposure and cerebral meningioma oncogenesis. The current epidemiological evidence has been consistent in observing increased associations with similar strengths of association among varying study populations and comparison groups; however, to date, these associations are correlative, and without the underlying mechanism, causation cannot be inferred.

### 4.4. Strengths and Limitations

The current results should be viewed in light of certain strengths and limitations. First, the current study utilized both an active and a non-active comparator as a comparison. Secondly, through the use of cancer controls derived from electronic health record data, the study was able to provide an anchor date that allowed for a more standardized determination of dMPA exposure status based on prior medical documentation. Further, the use of separate tumor control types allowed for the examination of whether the associations remained consistent across the control groups. As a main limitation, the current study is based on a single center and may not be generalizable to all populations. Research has suggested that there are regional differences in meningioma characteristics (e.g., size at presentation) and treatments by region [26]; thus, it is possible that the patient population in the current study is not reflective of all patient populations and that associations may differ from studies performed at other centers. That said, the use of electronic medical record data allowed for a more thorough dMPA exposure documentation from physician orders, prescriptions, and medication reconciliation rather than only from dispensed prescriptions, which was one of the limitations of Griffin [3]. Furthermore, results may be biased by residual confounding for two reasons. First, though a bootstrapping methodology was used to produce stable estimates of the association, the prevalence of dMPA exposure was low, preventing models from including more than a handful of covariates. Second, there are potential confounders that were either missing for a proportion of patients (i.e., body mass index) or were not available in the data (e.g., reproductive history and parity), preventing their inclusion in the models.

Regarding exposure measurement, due to the low dMPA exposure, particularly among cases, the associations should be interpreted with caution. In addition, the utilization of medical documentation for the determination of dMPA exposure could lead to dMPA exposure misclassification; however, the documentation included that from medication reconciliation during the patients’ cancer-related encounters, which includes an assessment of exposures that occurred outside of our institution. Further, there is no reason to expect the misclassification to be related to dMPA exposure status; thus, any bias would be towards the null, resulting in our associations being underestimations of the true association. Finally, the utilization of tumor controls may result in an exposure prevalence among the control group that is not representative of the general population. It has been estimated that 3% of all women have used dMPA [27], higher than the 1.1% prevalence of all controls combined in the current study. As a result, the observed increased associations may be biased by the lower dMPA prevalence among the control groups relative to the general population if the use (or lack thereof) of dMPA was related to the patient’s cancer diagnosis.

## 5. Conclusions

The current study adds to the body of literature reporting an increased association between depo medroxyprogesterone acetate and cerebral meningioma. This association has now been reported among three different studies: (1) A French study utilizing population controls selected from a French national health registry and intracranial meningioma cases who had surgery; (2) a study utilizing data from a database of women with employer-provided commercial insurance who sought medical care and included cases regardless of treatment; and (3) a single-center study utilizing controls with diagnosed non-meningioma cancer and meningioma cases regardless of treatment. Physicians may want to consider discussing with their patients the potential association between a cerebral meningioma diagnosis and the usage of dMPA.

## Figures and Tables

**Table 1:** ICD-O-3 topography and histology codes of cerebral meningioma cases and matched controls.

	Topography	Histology (in Order of Descending Frequency)
Cerebral meningioma cases	C70.0, C70.9	953 (100.0%)
Skin tumor controls *	C44.1, C44.2, C44.3, C44.4, C44.5, C44.6, C44.7, C44.9	872 (58.4%), 874 (37.7%), 877 (0.9%), 883 (0.7%), 824 (0.4%), 841 (0.4%), 970 (0.4%), 807 (0.2%), 820 (0.2%), 840 (0.2%), 878 (0.2%), 880 (0.2%), 912 (0.2%), 959 (0.2%)
Non-meningioma brain tumor controls *	C71.0, C71.1, C71.2, C71.3, C71.4, C71.5, C71.6, C71.7, C71.8, C71.9	944 (42.2%), 940 (23.5%), 938 (9.6%), 945 (8.0%), 950 (2.8%), 947 (2.4%), 968 (2.4%), 916 (2.0%), 939 (2.0%), 956 (2.0%), 942 (1.6%), 896 (0.4%), 912 (0.4%), 935 (0.4%), 936 (0.4%)
Breast tumor controls *	C50.0, C50.1, C50.2, C50.3, C50.4, C50.5, C50.6, C50.8, C50.9	850 (72.4%), 852 (21.0%), 821 (1.7%), 801 (0.9%), 820 (0.8%), 848 (0.8%), 814 (0.5%), 902 (0.4%), 823 (0.2%), 857 (0.2%), 853 (0.2%), 851 (0.1%), 854 (0.1%), 912 (0.1%), 800 (0.1%), 805 (0.1%), 825 (0.1%), 834 (0.1%), 880 (0.1%), 975 (0.1%)

* percent for controls is based on the number of unique persons across the 1000 bootstrapped samples.

**Table 2:** Comparison of characteristics of intracranial meningioma cases and matched controls.

			Tumor Control Type (ICD-O-3 Topography Code)		
	Cases (*N* = 241)	Controls (95% CI) *	Brain (C71) (95% CI) *	Breast (C50) (95% CI) *	Skin (C44) (95% CI) *
Mean Age, Years	59.6	59.3 (59.1–59.4)	58.3 (58.1–58.5)	59.7 (59.4–59.9)	59.5 (59.3–59.8)
Race, %					
White	58.6	78.8 (76.6–81.1)	77.6 (74.9–80.2)	63.7 (58.2–69.2)	95.3 (93.5–97.0)
Non-White	41.4	21.2 (18.9–23.4)	22.4 (19.9–25.1)	36.3 (30.8–41.8)	4.7 (3.0–6.5)
Rural–Urban Commuting Area, %					
Metropolitan	73.6	77.6(75.4–79.9)	73.7 (71.0–76.6)	81.4 (76.7–85.8)	76.6 (73.1–80.1)
Micropolitan	15.1	12.0 (10.2–13.7)	12.7 (10.1–15.0)	9.2 (5.9–12.6)	14.3 (11.2–17.3)
Small Metro/Rural	11.3	10.4 (8.7–12.1)	13.6 (11.5–15.9)	9.4 (5.9–12.9)	9.1 (6.6–11.3)
Insurance type, %					
Medicaid/Medicare	12.8	10.4 (8.6–12.3)	10.0 (8.2–11.9)	13.4 (9.2–17.6)	7.7 (5.6–9.9)
Commercial	71.4	78.6 (76.3–80.8)	75.6 (73.0–78.5)	75.7 (70.9–80.6)	83.7 (80.6–86.9)
Other	8.6	6.6 (5.2–8.0)	7.4 (5.7–8.9)	7.4 (4.6–10.5)	5.0 (3.1–6.9)
Uninsured	7.3	4.5 (3.4–5.6)	7.0 (5.2–8.4)	3.5 (1.7–5.9)	3.5 (2.2–5.2)
Elixhauser Comorbidities					
Mean Unweighted Score	2.3	1.7 (1.6–1.8)	2.9 (2.7–3.0)	1.4 (1.2–1.7)	1.2 (1.1–1.3)
Category, %					
0 Comorbidities	36.4	43.2 (40.6–45.9)	28.2 (25.4–30.8)	49.8 (44.0–55.5)	47.6 (43.4–52.0)
1 Comorbidity	15.9	24.9 (22.5–27.1)	15.4 (13.2–17.7)	24.7 (19.9–29.7)	32.2 (28.2–35.9)
2 Comorbidities	18.0	11.8 (10.0–13.5)	17.0 (14.8–19.1)	10.7 (6.7–14.6)	9.1 (6.8–11.6)
3 Comorbidities	11.3	6.9 (5.6–8.2)	12.0 (9.8–14.1)	5.6 (2.9–8.7)	4.4 (2.6–6.1)
≥4 Comorbidities	18.4	13.2 (11.7–14.7)	27.3 (25.6–30.1)	9.2 (5.9–12.9)	6.7 (4.7–8.6)

* estimated from 1000 bootstrap samples; reported as the mean values across the samples and the 95% CI as the 2.5th and 97.5th percentiles for the lower and upper limits, respectively.

**Table 3:** Crude and adjusted odds ratios *,† (ORs) and 95% confidence intervals (CIs) for the association between depot medroxyprogesterone acetate exposure (compared to active ‡ comparator or non-active comparator) and intracranial meningioma.

	Exposure, %		dMPA Exposure vs. Comparator Exposure, OR (95% CI)		
	Controls (95% CI)	Cases	Crude	Age- and Race- Adjusted	Fully Adjusted
ANY EXPOSURE					
dMPA	1.1 (0.6–1.7)	2.9	-	-	-
Levonorgestrel/norethindrone	5.4 (4.2–6.8)	6.7	2.08 (1.17–4.26)	1.25 (0.61–2.96)	1.16 (0.54–2.95)
Non-active	93.4 (92.0–95.0)	90.4	2.75 (1.69–5.56)	1.95 (1.07–4.26)	1.91 (0.99–4.50)
ONE YEAR PRIOR EXPOSURE					
dMPA	0.4 (0.2–0.8)	2.9	-	-	-
Levonorgestrel/norethindrone	3.8 (2.8–4.9)	5.4	5.52 (2.36–14.09)	3.51 (1.37–9.28)	3.27 (1.19–9.89)
Non-active	95.8 (94.7–96.9)	91.6	8.56 (3.91–19.01)	6.73 (2.88–16.07)	6.71 (2.69–18.09)
TWO YEARS PRIOR EXPOSURE					
dMPA	0.7 (0.3–1.1)	2.9	-	-	-
Levonorgestrel/norethindrone	4.3 (3.2–5.5)	5.4	3.58 (1.88–8.00)	2.27 (1.03–5.76)	2.13 (0.95–6.30)
Non-active	95.0 (93.8–96.2)	91.6	4.83 (2.72–9.45)	3.79 (1.93–8.92)	3.73 (1.75–9.82)

* based on a conditional logistic regression; † estimated from 1000 bootstrap samples; OR reported from the mean of the estimated; OR across the samples and the 95% CI as the 2.5th and 97.5th percentiles for the lower and upper limits, respectively; ‡ Active comparator is use of medications including norethindrone (alone or in combination) or combinations of levonorgestrel adjusted for age, race, and urban/rural classification of residential ZIP code, insurance type, and unweighted Elixhauser comorbidity score.

## Data Availability

Due to the nature of the data (electronic health record data), data are not made available for distribution.

## Abbreviations

The following abbreviations are used in this manuscript:

MPA	Medroxyprogesterone acetate
dMPA	depot Medroxyprogesterone acetate
RUCA	Rural–Urban Commuting Area
OR	Odds ratio
CI	Confidence interval
